# Retrograde Approach to a Diverticular Kidney Stone through a Vesicoureteral Cohen's Reimplantation: A Novel Surgical Technique

**DOI:** 10.1155/2013/247528

**Published:** 2013-03-14

**Authors:** Fahd Khalil, Mohamed Tligui, Olivier Traxer

**Affiliations:** Urology Department, Tenon Hospital, 4, rue de la Chine, 75970 Paris Cedex 20, France

## Abstract

Cohen's technique is the standard treatment of vesicoureteral reflux in children. Its disadvantage is still the classic difficulty in subsequent retrograde ureteral access, requiring the use of percutaneous techniques in the treatment of kidney stones. We describe a novel surgical technique for retrograde catheterization of an adult ureter by a flexible ureterorenoscope, thereby facilitating the treatment of a symptomatic diverticular kidney stone. We compare our technique to other methods described in the literature.

## 1. Introduction

The retrograde access through Cohen's reimplantation for the minimally invasive treatment of an intradiverticular symptomatic kidney stone combines two major anatomical difficulties [1, 2]. Our technique allows resolving this anatomical problem by the use of a flexible ureterorenoscope coupled with the Holmium-YAG laser.

## 2. Observation

**Figure 1:**
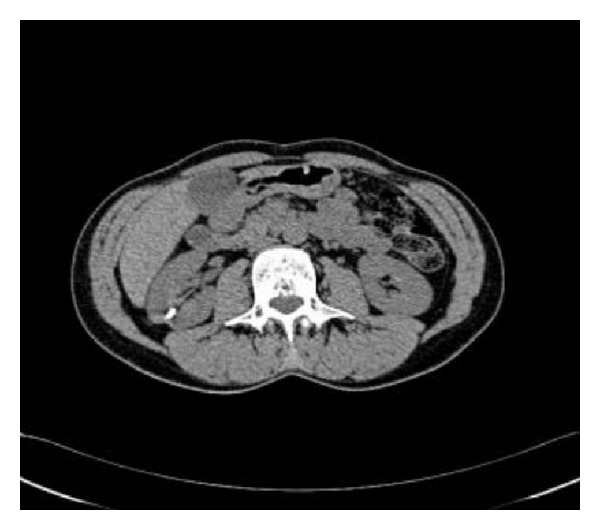
CT scan. Right calyx stone.

**Figure 2:**
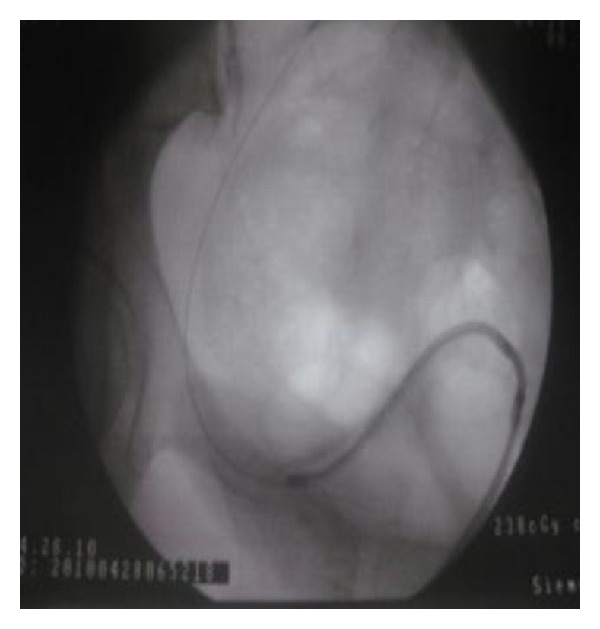
Ureteral meatus catheterization.

**Figure 3:**
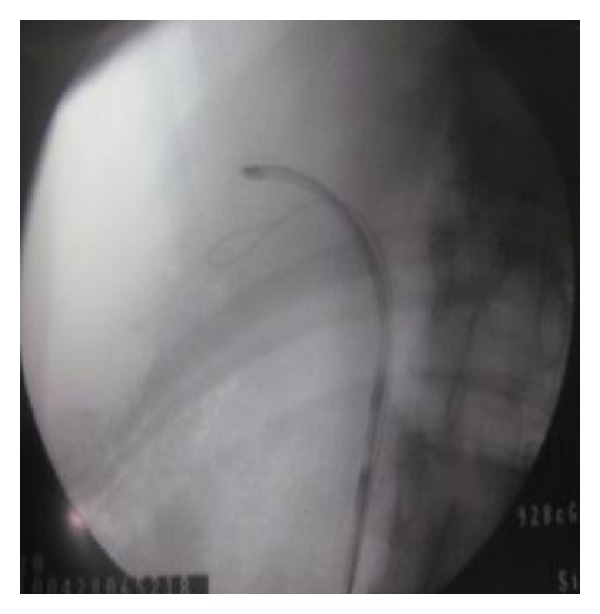
Ureteroscope in the upper tract.

**Figure 4:**
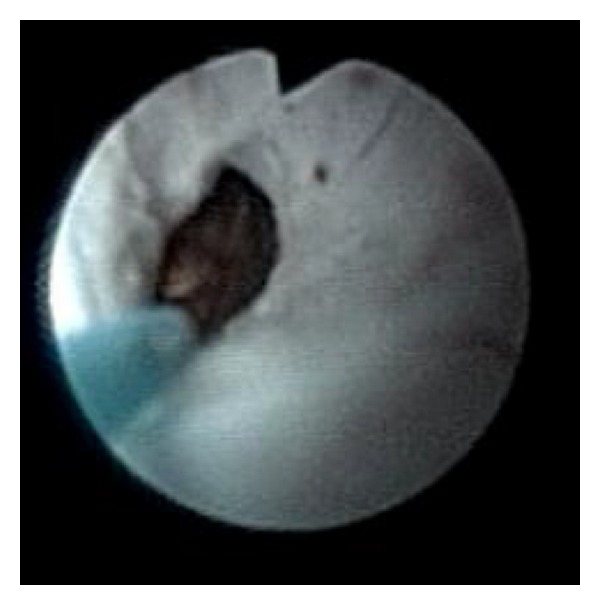
Laser incision of the diverticular neck.

A 30-year-old man received at the age of 5 years a surgical management of vesicoureteral reflux symptoms consisting on a Cohen's type ureterovesical reimplantation. The postoperative course was uneventful with good clinical improvement. His current story evolves from six months with gradual onset of lumbar pain despite a regular intake of analgesics. The intravenous urography showed a lower calyceal calculation of 12 mm inside a diverticulum confirmed by the CT scan imaging (Figure 1). Our technique has the characteristic of the use of a flexible ureteroscope for locating the ureteral meatus of the reimplanted ureter, which requires a certain experience in handling the instrument. After the catheterization of the ureteral meatus (Figure 2), two 0.035 inch curve guide wires are inserted to the kidney cavities, one of which is used for rising the flexible ureterorenoscope and performing a retrograde ureteropyelography (Figure 3). We proceed to the identification of the diverticular neck under fluoroscopic and visual guidance and an in situ stone's fragmentation by the holmium: YAG laser (Figure 4). The final result was satisfactory with a complete stone fragmentation.

## 3. Discussion

A systematic medline database review found only exceptional descriptions of surgical techniques of retrograde catheterization of the Cohen [1, 3]. To our knowledge, we describe the fourth retrograde ureteral access technique. Lamesch [3] used a suprapubic bladder trocar to have an ureteral access, but with a 80° angle and a major risk of ureteral injury. Argueso et al. catheterised the ureteral meatus with a COBRA 5Fr probe and a 0.035 inch guide wire [4]. Wallis et al. [5] used a curved 4fr guide wire traditionally used in the interventional imaging to cannulate the ureteral meatus. In our technique, the retrograde use of a flexible ureteroscope has overcome the difficulty of the 90° angulation between the cystoscope and the meatus of the reimplanted ureter. It was also an efficient approach to the upper urinary tract stones due to the inefficiency of ESWL in such locations, as well as a minimally invasive management by replacing the PCNL in stones less than 2 cm allowing diverticular neck incision by the Holmium-YAG laser.

## 4. Conclusion

Flexible ureteroscopy transformed the approach of a potentially complicated anatomical procedure into a simple endourological one. It is an interesting minimally invasive technique in Cohen's type reimplantation for the treatment of the diverticular complicated stones.

## References

[1] Lusuardi L, Hruby S, Jeschke S, Zimmermann R, Sieberer M, Janetschek G (2011). A new technique for retrograde flexible ureteroscopy after Cohen cross-trigonal ureteral reimplantation. *Urologia Internationalis*.

[2] Lechevallier E, Saussine C, Traxer O (2008). Prise en charge des calculs des diverticules caliciels rénaux management of stones in renal caliceal diverticula. *Progrès en Urologie*.

[3] Lamesch AJ (1981). Retrograde catheterization of the ureter after antireflux plasty by the Cohen technique of transverse advancement. *Journal of Urology*.

[4] Argueso LR, Kelalis PP, Patterson DE (1991). Strategies for ureteral catheterization after antireflux surgery by the cohen technique of transverse advancement. *Journal of Urology*.

[5] Wallis MC, Brown DH, Jayanthi VR, Koff SA, Caione P (2003). A novel technique for ureteral catheterization and/or retrograde ureteroscopy after cross-trigonal ureteral reimplantation. *Journal of Urology*.

